# Perceptions of Audio Computer-Assisted Self-Interviewing (ACASI) among Women in an HIV-Positive Prevention Program

**DOI:** 10.1371/journal.pone.0009149

**Published:** 2010-02-10

**Authors:** Larissa J. Estes, Linda E. Lloyd, Michelle Teti, Sheela Raja, Lisa Bowleg, Kristi L. Allgood, Nancy Glick

**Affiliations:** 1 School of Public Health, University of Texas, Houston, Texas, United States of America; 2 School of Public Health, Drexel University, Philadelphia, Pennsylvania, United States of America; 3 College of Dentistry, University of Illinois at Chicago, Chicago, Illinois, United States of America; 4 Sinai Urban Health Institute, Sinai Health System, Chicago, Illinois, United States of America; University of Toronto, Canada

## Abstract

**Background:**

Audio Computer-Assisted Self Interviewing (ACASI) has improved the reliability and accuracy of self-reported HIV health and risk behavior data, yet few studies account for how participants experience the data collection process.

**Methodology/Principal Findings:**

This exploratory qualitative analysis aimed to better understand the experience and implications of using ACASI among HIV-positive women participating in sexual risk reduction interventions in Chicago (*n* = 12) and Philadelphia (*n* = 18). Strategies of Grounded Theory were used to explore participants' ACASI experiences.

**Conclusion/Significance:**

Key themes we identified included themes that could be attributed to the ACASI and other methods of data collection (e.g., paper-based self-administered questionnaire or face-to-face interviews). The key themes were usability; privacy and honesty; socially desirable responses and avoiding judgment; and unintentional discomfort resulting from recalling risky behavior using the ACASI. Despite both positive and negative findings about the ACASI experience, we conclude that ACASI is in general an appropriate method for collecting sensitive data about HIV/AIDS risk behaviors among HIV-positive women because it seemed to ensure privacy in the study population allowing for more honest responses, minimize socially desirable responses, and help participants avoid actual or perceived judgment.

## Introduction

Measures of health risk behaviors often function in public health research without a “gold standard” measurement of comparison due to biases imposed by self-reporting. Reliability studies have aimed to improve the accuracy of self-reports through modified methods of data collection, such as self-administered versus interviewer-administered instruments [1]. Research indicates that some methods of data collection may increase disclosure and minimize socially desirable responses. One such method, Audio Computer-Assisted Self interviewing (ACASI) is a tool used in data collection to collect sensitive data about health and risk behaviors. ACASI allows the respondent to use headphones connected to a laptop or desktop computer to listen to instructions, questions, and responses that have been digitally recorded onto an ACASI program while corresponding text is displayed on the computer monitor [2]. Respondents are able to enter his or her responses directly into the computer using a keyboard, touch screen or mouse [2].

ACASI allows those who have low-levels of literacy to rely upon the audio component of the survey; whereas those who are hearing impaired can rely on the text displayed on the computer monitor [3]. ACASI provides anonymity, allowing for presumed more honest responses to questions of a personal or sensitive nature, or those that may be considered as socially undesirable [2]. ACASI allows complex surveys to be standardized which may be difficult for interviewers to administer during a face-to-face interview because of the complexity of questions and skip patterns ([4]. The quality of the data collected using the ACASI is enhanced because data is collected directly from the participant, minimizing errors in transcription and data entry [2]. Consistency checks can also be auto-programmed into an ACASI program to minimize additional human error.

Data collection methods may vary in their ability to accurately capture data. The most popular modes of data collection are paper-based self-administered questionnaires, interviewer-administered telephone interview, and face-to-face (FTF) interviews. Unbiased measurement is important. Biased measurements can affect the quality of care from interventions derived from various data collection methods. Kim, et al. (2008) found in their comparison of 3 methods for gathering data (face-to-face interviews, paper-based self-administered questionnaire, and ACASI) that participants would reveal more sensitive information on the ACASI compared to the paper-based self-administered questionnaire [5]. Another study found that the ACASI elicited higher reports of risk behaviors [6]. A study assessing response bias among STD clinic patients found that among respondents, women were more likely to admit to certain risk behaviors with ACASI compared to face-to-face interviews [7]).

HIV-positive women can experience the stigma of disease, gender, poverty, and race/ethnicity [8]–[9]. A qualitative research synthesis of research on HIV-positive women by Sandelowski, Trimble, Woodard & Barroso (2006) found that women feared and experienced negative social effects, including social rejection, discrimination and violence [10]. Women often dreaded, anticipated, experienced and adapted their lives to the blatant and discrete stigmatization they attributed to their HIV serostatus in both intimate and distant relationships [10]. Consequences of stigma affect access to financial independence, care, knowledge and social connectedness [11]; as well as induce depression, anxiety, loneliness and decreased self-esteem [12]. To avoid the additional stigma associated with HIV and associated risk behaviors, ACASI may allow women who experience stigma and biases to freely disclose behaviors and experiences that may otherwise not be disclosed because of the risk of social isolation and increased discrimination [7], [13].

There is limited research that accounts for a woman's experience with ACASI; only three of the studies reviewed for this paper used only women in their ACASI-related research [14]–[16]. Despite limited research on the experience of women when using ACASI, existing research indicates that ACASI is beneficial for research, including: additional privacy in the absence of an interviewer [13], [15], [17]–[18]; more truthful responses [13]–[15], [18]; minimizing socially desirable responses [16], [19]; alleviation of differences in literacy [17]; and ease of questionnaire navigation. For researchers, ACASI provides immediate access to data. Outside of published research indicating that participants prefer ACASI due to increased privacy [16]–[18] and confidentiality [15], we know little about how participants, especially women, experience computerized surveys like ACASI.

A participant's experience is particularly pertinent for studies among women at risk for and living with HIV given that the majority report low income and educational opportunities and may not have had previous experience using a computer. The documented and prospective research benefits to ACASI make participant experience worthy of further exploration. The aims of this analysis were to explore the experience of using ACASI among HIV-positive women in Philadelphia and Chicago who participated in the Health Resources and Services Administration (HRSA)–funded *Prevention with HIV-Infected Persons Seen in Primary Care Settings* or *Prevention with Positives* Initiative [20] and to explore the implications of using ACASI for research.

## Methods

### Ethics Statement

The following methods were approved by both the Sinai Health System and Drexel University Institutional Review Boards (IRB) in 2004. Each demonstration site recruited its own patients. When a patient demonstrated interest in participating, she received the IRB approved written consent form. The consent form was reviewed by the patient with the recruiter and signed by the patient indicating their consent to participate.

### Study Setting

The Philadelphia and Chicago demonstration sites were selected for inclusion in this qualitative study from the 15 HRSA-funded *Prevention with Positives* demonstration sites. The Philadelphia and Chicago sites were identified as having both individual-level and peer-administered interventions [20]. In addition, both the Philadelphia and Chicago demonstration sites also had large low-income, Hispanic and African-American patient populations [21]–[22]. Many of the other demonstration sites used interventions administered by a primary care provider or intervention specialist and are detailed in the September 2007 supplement published by AIDS and Behavior dedicated to this HRSA Special Project of National Significance.

The Philadelphia demonstration site created the *Protect and Respect* intervention program. The goal of the *Protect and Respect* program was to decrease sexual behavior that placed HIV-positive women at risk for STIs and others at risk for HIV transmission [21]. The program's intervention group received HIV prevention messages incorporated during their routine medical visits with clinicians, a group-level intervention (GLI) delivered by a health educator and a peer led support group [21]. The GLI consisted of five weekly education and skill building session [21]. The peer led support group were weekly support groups that featured educational topics such as re-infection, healthy and unhealthy relationships, and strategies for living with HIV [21]. The comparison or control group received brief messages delivered by a health care provider during routine medical visits [23].

In Chicago, the program development team created “Treatment Advocacy Program Intervention-Sinai” (TAP-Sinai). The primary goal of TAP-Sinai was to help HIV-positive men and women increase their adherence to medication regimens and sexual safety skills [22]. TAP-Sinai used multiple one-on-one education sessions with an HIV-positive peer from the community [22]. TAP-Sinai's intervention group received four mandatory individually tailored modules, guided by a peer educator [22]. Modules included basic information on HIV, HIV medication adherence, coping, and sexual safety behaviors [22]. The TAP-Sinai control group received usual care which consisted of a medical appointment, case management and medical adherence counseling.

### Recruitment Procedures

The Partnership Comprehensive Care Practice (PCCP) in Philadelphia was the site for *Protect and Respect*. The PCCP provides comprehensive and integrated HIV services to more than 1300 adult patients annually; 32% of whom are women [23]. The recruitment team included two HIV-positive Peer Educators (PEs) and three Research Assistants (RAs) [23]. PEs and RAs recruited women from the PCCP's waiting room using flyers to initiate discussions with women who were waiting for their regularly scheduled medical visits [23]. To be eligible to participate, women had to be at least 18 years old, HIV-positive for at least 6-months, and English-speaking [23]. The Drexel University IRB approved the project and study procedures.

In Chicago, Mount Sinai Hospital's Infectious Disease (ID) clinic was the study site for TAP-Sinai. The ID clinic serves about 400 HIV-positive individuals largely composed of low-income, African American (80%) and Hispanic (20%) patients [22]. Forty-seven percent of the ID clinic patients are women. The recruitment team consisted of two PEs and one RA. The PEs and RA recruited participants at the ID clinic after they were seen by a physician. The physician introduced the study and a PE or RA to the patient. The recruitment team screened all patients who were approached for eligibility. To be eligible to participate, women had to be at least 18 years old, HIV-positive for at least 3-months, English-speaking, attended at least one clinic visit in the prior 12-months, and mentally able to provide informed consent [22]. This project and all materials were approved by the Sinai Health System IRB.

Both *Protect and Respect and TAP-Sinai* interventions included English speaking participants because all 15 sites participating in the HRSA demonstration project did not have a Hispanic population and the ACASI contained a dominant number of core questions. Costs to translate the ACASI and hire Spanish-speaking trained interviewers would have significantly increased costs. Participants from TAP-Sinai and Protect and Respect were randomly assigned to intervention and control arms of each site's intervention program.

### Participants

In Philadelphia, the total *Protect and Respect* sample included 185 women with HIV/AIDS between the ages of 20 and 70 (*M* = 40, *SD* = 8.5) [23]. The sample was predominantly racial/ethnic minorities (85%) and low income (76% of the sample reported annual incomes of $10,000 or less) [23]. *Protect and Respect* participants were living with HIV for a range of 2 to 20 years (*M* = 9, *SD* = 5) [23]). The qualitative sample for this study included 18 women who received the in the *Protect and Respect* intervention. Interviewees were 28 to 47 years of age (*M* = 42, *SD* = 7) and were predominantly low-income (61% reported annual incomes of $10,000 or less); living with HIV for a range of 2 to 10 years (*M* = 9, *SD* = 5).

In Chicago, the total TAP-Sinai sample included 79 women and 94 men. The average age for women and men were 44 and 41 years, respectively (M = 43 SD = 10). The average years living with HIV was 7.2 years for women and 9.2 years for men (M = 8.3, SD = 6.0). The TAP-Sinai sample included predominately racial/ethnic minority women and men (94% for both women and men). Over 68% of respondents reported an income of ≤$10,000 (65% for women and 71% for men). The qualitative sample from TAP-Sinai included 12 women. Interviewees were 30 to 58 years of age (*M* = 44, *SD* = 8) and were predominantly low income (50% reported annual incomes of $10,000 or less). They were living with HIV for a range of three months to 19 years (*M* = 8, *SD* = 6).

### Measurement

At the *Prevention with Positives* demonstration sites, researchers administered a questionnaire using an ACASI at baseline, 6, 12, and 18 months to measure participant risk behaviors [20], [23]. ACASI was the primary method of data collection. The ACASI included specific questions about participants' sexual partners; oral, vaginal, and anal sex practices; condom use; self efficacy to solve problems; attitudes towards HIV prevention; drug and alcohol use; views on health issues and demographic information. Each ACASI questionnaire lasted 30 minutes.

To gain a more in-depth understanding of the experiences of women using ACASI, researchers in both sites conducted a nested qualitative study through semi-structured interviews with a subsample of women. In Philadelphia, two female RAs conducted 18 interviews with women. In Chicago, one female RA conducted interviews with 12 women. The total number of women interviewed for this study was 30. Interviews at both sites lasted approximately one hour and were audio-taped. Women in Philadelphia received a $10 cash incentive, whereas women in Chicago received a $30 gift certificate.

### Qualitative Interviews

For the qualitative interviews, both sites approached women who received the *Protect and Respect or TAP-Sinai* interventions at their respective sites through telephone calls asking them if they wanted to complete a post intervention interview. Not all women were contacted to participate in the post-intervention interview because some did not have a working phone or had moved. This method was used until a sufficient number of participants agreed to participate in the interviews. The sample size of 18 women from Philadelphia and 12 women from Chicago is consistent with the “15±10” metric for qualitative interview studies [24]. Interview guides were tailored to each site; however the interview guides featured core questions addressing participants background/motivation to participate, intervention experiences (education and peer support groups), the impact of group (on women and their behaviors), perspectives on HIV prevention, and the experience with and honesty on the ACASI. Each site was able to add site-specific questions however the number of questions the sites were allowed to add was limited. The questions were developed by the multi-disciplinary team at University of California, San Francisco AIDS Policy Research Center which served as an evaluation and support center for this HRSA initiative with input from all participating demonstration sites [20], [25]. The nested-qualitative study was not intended to seek validity in themes but to explore themes or concepts related to the participant's experience using ACASI. To improve the reliability of the qualitative interview, the interviews were tape-recorded and transcribed.

### Analysis

This study was an exploratory study, exploring the experiences of women using ACASI. Researchers in Philadelphia and Chicago transcribed audio-taped interviews and edited them to remove personal identifiers. To maximize reliability, the first and third authors read the transcripts thoroughly multiple times to become acquainted with the data and developed a code book of key themes to guide analyses of participants' experiences with the ACASI. The data was imported into *Atlas.ti*, a qualitative data analysis software package. The data was coded and analyzed using two strategies derived from Grounded Theory, coding and memo writing. Grounded Theory is rooted in the cyclical process of collecting data, analyzing it, and developing a provisional coding scheme [26]. Coding progressed in two stages: the intense initial and focused coding of interview transcripts, followed by the discussion of the text that researchers had interpreted or coded differently until 100% consensus was reached [27]. Coding can generate a long list of concepts which is then categorized into more sophisticated schemes by grouping those concepts that appear to be related to a similar phenomenon. The trustworthiness of our analyses was assessed using four criteria: prolonged engagement with data, credibility, transferability, and confirmability [28]–[29]. As the analysis progresses, theory emerges both inductively and deductively [26]. Participants are described below using pseudonyms (to ensure confidentiality), age, and intervention site (Philadelphia or Chicago).

## Results

We set out to explore the experience of using ACASI among HIV-positive women. As we anticipated, our findings were applicable specifically to using ACASI and/or computerized surveys. The following themes will be explored in further detail below: usability, privacy and honesty, and the absence of personal interaction. We also identified three themes that were applicable to conducting research with this population in general and not just to computer surveys: socially desirable reporting, being honest to contribute to research, and the impact of unintentional harm from recalling risky behavior. The results are organized by (1) ACASI-specific results and (2) results that can be applied to other methods of data collection.

### ACASI-Specific Results

#### Usability

We defined usability as the participants' experience responding to the questionnaire in terms of efficiency and overall satisfaction with the experience. The usability appears to be ACASI-specific because it results from participants interacting directly with the ACASI to complete the computer based, self-administered questionnaire, not responding to questions asked by an interviewer. Participants generally found the ACASI easy to use; however two women noted the apparent repetitiveness of some of the questions administered via ACASI:

When [I] got to the part about how many partners you had, and do they have HIV or was they negative or positive…[I] was about ready to take the whole computer [and] pull it out the wall, cause [it] kept going back and forth to the same questions (Daisy, 47 - Philadelphia).

Likewise, Rita described the ACASI's questions as being repetitive, y*et* alluded to it being “okay” once she became comfortable with the process of completing the ACASI:

It just asked me the same thing over and over again. You know, are you heterosexual, are you bisexual, are you a drug user, are you an alcoholic, you know stuff like that. How long have you had the virus, when did you get the virus, and stuff like that. It was okay after I got the hang of it (Rita, 50 - Philadelphia).

To measure participant risk behaviors, participants completed the ACASI at baseline, 6, 12 and 18 months. Asking the same questions over a span of 18 months may seem repetitive to respondents but was necessary to standardize questions to measure change in risk behaviors. Though it took Rita some time to become comfortable with the process of answering questions using ACASI, she also acknowledged that ACASI was faster compared to completing a paper-based self-administered questionnaire:

If we had to write and answer all of those questions on paper we would have been there a little longer. It was a lot of questions, but it was nice. I got the hang of it now.

The repetitiveness could also have been minimized using face-to-face (FTF) interviews. In a FTF interview, the personal interaction with an interviewer allows for the clarification of questions, the stressing of the importance of answering questions, and reemphasizes on the need for repetitive questioning.

#### Privacy and honesty

Participants believed that using ACASI ensured confidentiality and provided privacy. Using the ACASI to complete a questionnaire may have made disclosure of risk behaviors easier and allowed for honest responses among participants in comparison to other survey methods such as self-administered, paper-based self-administered questionnaire or a FTF interview. Marlene remarked, “It's personal, you are the only one on the computer putting the information in…nobody [knows] exactly what you are putting down.” (Marlene, 34 - Philadelphia) Similarly, Leesa commented, “You'd probably get a more serious and honest answer that way than you would face-to-face.” (Leesa, 47 - Philadelphia)

Many participants indicated that it was easier to disclose their risk behaviors to a computer than to an interviewer in a FTF interview. Anita acknowledged this saying, “Yeah…because some people won't sit with someone and really tell them [the] truth. They'll say anything, but they'll be more open to a machine before they be comfortable with somebody real.” (Anita, 47 - Philadelphia) A few women indicated that in order to feel comfortable disclosing in a FTF interview the interviewer would have to gain their trust. Alexa recalled, “It was good because when you first get a person, if [you] don't want to talk to somebody, [you won't] really want to answer those questions verbally.” (Alexa, 43 - Philadelphia) Rochelle acknowledged that the process of disclosure can take some time if disclosing risk behaviors to others:

Cause some people don't like to talk about some [things]. It takes a while, like I said, like [in] our women's group. A lot of times when people come in they won't say nothing for a minute and then they [start] talking about what they really wanted to talk about last week, but they was scared [to talk about it]. [Be]Cause you know it's a process, it takes a while (Rochelle, 51 - Philadelphia).

Some participants felt that the ACASI provided privacy that allowed them to avoid perceived judgment by an interviewer. Trina noted:

I've been strictly doing this on the computer, you know. So the computer and I have been making love, interacting. You know what I'm saying? Not you and I, so why would I give you the opportunity to know me intimately when all you've asked me was how was my day and how long you're going to be here. Hell, I don't know you like that. (Trina, 36 - Chicago)

Several women were concerned with being judged by an interviewer and acknowledged ACASI as a mechanism to ensure confidentiality and avoidance of perceived judgment by an interviewer. Rochelle acknowledged this saying, “The computer is not gonna judge you, you know what, I mean?” (Rochelle, 51 - Philadelphia) Fana agreed, saying, “You don't have to think what the person thinks of you or what is in the back of their mind (Fana, 44 - Philadelphia).” Susan felt similarly about avoiding judgment, “Right, because, you know, you say certain things, you answer certain questions… [and] you don't know how people [are] going to look at you. That computer can't judge you or look at you (Susan, 44 - Chicago).” The privacy that ACASI provided our participants appeared to allow them to provide more honest answers.

Overwhelmingly, participants in Chicago and Philadelphia said that they felt that ACASI was a good way to elicit honest answers about risk behaviors. Shandra discussed the ACASI's privacy as allowing her to feel “comfortable;” and to be honest when completing ACASI:

I was comfortable. Yeah, that's very good. [Be]Cause you're not talking to nobody, you're just on the computer. And then your name ain't even on here. So you could be honest. You ain't got to lie about nothing. That's why I told y' all the truth. I ain't got no sex partners. No, I don't do no drugs. I tell y'all all the truth (Sandra, 31 - Chicago).

#### Absence of personal interaction with ACASI

A couple of respondents missed the personal interaction from a FTF interview while using ACASI. For example, Glenda mentioned that she was not able to receive feedback from the computer while Lena noted that she was unable to provide context to describe her responses while answering the ACASI questions. Glenda said that the computer was “impersonal”. She elaborated further:

Like I'm talking to a machine and I'd rather talk to a person. Somebody else could feel different you know…Yeah, it was okay but I probably want a person ‘cause then I could get some input if I was talking to a regular person. I could get feedback (Glenda, 44 - Philadelphia).

Lena discussed how a FTF interview allowed her as a respondent to provide context to a response, specifically acknowledging that she would not be able to provide that to a computer:

If I tell you something I want to be able to explain my reasoning behind it… With a computer, I can't explain why I answered that question yes. And then because I suffer from anxiety, I don't want to think that you have to decipher what I meant by that. (Lena, 30–Chicago)

Though some respondents missed the personal interaction that FTF interviews provided, ACASI appeared to provide more privacy; allowing respondents to provide more honest answers.

### Results Applicable to All Methods of Data Collection

#### Socially desirable responses

Participants said that they were honest on the ACASI throughout data collection, but admitted that it was likely that other women may have provided dishonest answers in order to maintain socially desirable norms, protect their privacy, or avoid fear or embarrassment with disclosure. The provision of socially desirable responses could happen in other methods of data collection (e.g. paper-based self-administered questionnaires and FTF interviews) and is not completely specific to ACASI. Thalia addressed this phenomenon, “I don't know because some people might just answer what they think you want to know, what you want them to say.” (Thalia, 35 - Philadelphia)

Trina acknowledged that high risk behaviors that are socially undesirable responses, such as anal sex, may not be disclosed to an interviewer but may be disclosed to ACASI:

Especially about anal sex because lot females aren't going to really tell you that they take it in the back…You know… It's embarrassing. I mean–it is. It's–most people you'll hear them say, ‘I don't do that. I don't do that.’ But when you ask them [if] they want some drugs they're going to do it. But they'll lie and say they don't [when asked by an interviewer]. I have said that I haven't done it, but I have…Not on the survey but to people…‘I don't do that.’ But then when you're on the computer you can put in there what, you know - the truth. (Trina, 36 - Chicago)

Other participants recognized the necessity of providing honest answers and its impact on the outcome of research.

#### Being honest to make contributions to research

Participants acknowledged the importance of providing honest answers because they understood that the data would be used to make important conclusions about HIV prevention. Paula stated:

Yeah, yeah, because nobody's looking and they're not putting a name to it…Yeah I think you get honest answers that way… Because it might help [the researchers] in the future, like maybe coming up with a cure for it or something (Paula, 40 - Philadelphia).

Likewise, in Chicago, Lena directly addressed the importance of honest answers to provide validity to the data, despite her fear to address questions of sexual assault directly:

Maybe that could have helped somebody else, because when I wrote down that I had just had sex by force, and then those questions came up about abuse, [it was] the perfect time for me to [answer] those questions [incorrectly]… That right there doesn't give validity to my answers when you guys get ready to [use] the data…only because I was scared to answer them (Lena, 30 - Chicago).

Despite the stipulation to provide honest answers, questions that force respondents to recall experiences and behaviors, may also cause harm to the respondent, despite the potential contribution to research.

#### Unintentional harm from recalling risky behavior

Several women acknowledged that questions administered by ACASI forced them to recall past behaviors that many respondents may have wanted to “forget” or not “re-live.” For example, Lena found recalling her past risk behaviors difficult when using the ACASI:

[W]hen I–when I first saw it [the question]because I wasn't in a relationship and I wasn't being abused–I haven't been abused in a long time, it was almost like opening up Pandora's Box for me…You know, almost like, ‘Why would they ask–what does this have to do with HIV and AIDS’, you know. ‘Oh, well, we're going to fake this, we're going to skip, skip, skip.’ You know, ‘No, no, no, no. Okay. Stop asking me. No, no, no, no.” (Lena, 30 - Chicago)

Though ACASI allowed women to be more honest, completing the ACASI was a difficult task without having someone (an interviewer) to support the recollection and processing of the past behavior. Brenda acknowledged that recalling and disclosing past risk behaviors can be in general very difficult; however disclosure was necessary: “I didn't find it difficult, you know some questions are deep, you know, and (unclear) It might bother you, but you gotta go through it, you gotta do it (Brenda, 41 - Philadelphia).” Respondents also feared harm from perceived judgment of an interviewer. Daisy noted, “…for me it's hard talking about it to somebody, so the computer, I ain't got to talk back to it, all I gotta do is push buttions…I ain't gotta worry about how they look at me.” Recalling past risk behaviors can be traumatic but not completely avoidable no matter the method of data collection.

## Discussion

In our analysis, we found that women described experiences that related specifically to using the ACASI and those that could also be applied to all methods of data collection with this population. The privacy ACASI affords allowed for more honest answers and the avoidance of perceived judgment from an interviewer in our study sample, although some women did note missing the personal interaction with a FTF interviewer. Though not specific to ACASI but also applicable to other methods of research, ACASI also did not prevent the provision of socially desirable responses and possibly caused unintentional emotional harm from the recollection of risky behavior or experiences. Respondents recognized the importance of being honest on the ACASI to affect research outcomes.

Results from this exploratory qualitative analysis supports two results from previous research on the use of ACASI: privacy and honesty. In this study, as in much of the published ACASI literature, respondents reported that ACASI ensured privacy [13], [16]–[18]. This finding suggests that ACASI's ability to ensure privacy may prevent women from experiencing perceived judgment from an interviewer and may diminish socially desirable reporting and thus, inaccurate data. ACASI appeared to be a good mechanism to elicit honest answers from participants and minimize the provision of socially desirable responses. This finding is consistent with findings from van de Wijert (2000), Metzger et al. (2000), Jones (2003), and Kurth et al.(2004) [13]–[15], [18]. When discussing honesty as related to ACASI, participants acknowledged that honest answers can affect the validity and quality of the research. This analysis further adds to the body of research supporting ACASI as an effective mechanism for ensuring privacy and eliciting honest responses from participants, particularly for women.

Although we sought out to explore women's experiences with ACASI, this analysis adds to a limited body of research about women's experiences with ACASI and adds to the larger body of research exploring the experience of women with other methods of data collection. The present analysis suggests three concepts not previously highlighted in the ACASI literature: 1) unintentional harm or discomfort; 2) acknowledgment of contribution to research; and 3) the noted absence of the participant-researcher relationship with ACASI. Of the three concepts are described below, only one appears to be exclusively ACASI-specific, the absence of the participant-research relationship.

Our findings suggest that participants may have experienced unintentional harm or discomfort while completing the ACASI, which prompted them to recall painful memories. Walker et al. (1997) found that female trauma survivors who reported unanticipated distress were more likely to have past trauma exposure and have high overall distress/symptom scores [30]. Elliot & Briere (1995) considered that among the general population recovered memories of child sexual abuse did not produce generalized distress but were more associated with symptoms of posttraumatic stress and self difficulties [31]. Though participants learn about potential risks and benefits of participation through reviewing informed consent forms and researchers anticipate risks and benefits, both are unable to completely predict discomforts and unintentional harm generated through any data collection technique. ACASI both instigated the recollection of memories (pleasant and unpleasant) and induced reflection upon those memories. Our findings suggest that the content of an ACASI-administered questionnaire may create or reinforce anxiety, causing unintentional harm or discomfort. Explorations of participants' risk behaviors through ACASI can lead to recollections of painful, upsetting memories, and even repressed memories. Paper-based self-administered questionnaires have also been found to cause unintentional harm or discomfort during the process of data collection [32]–[33]. Griffin et al. (2003) found that participants reported that computer-based questionnaires made them feel less reserved in their response [34].

Researchers must identify the possibility of harm or discomfort and provide access to post-interview support, no matter the method of data collection [35]. Potential participants should be informed of the areas covered by the questionnaire in advanced and be provided enough information in a sufficient, clear manner during the consent process [33]. Questionnaires administered by ACASI must be designed to minimize any adverse effects including anxiety that may arise during the ACASI session [32]. Griffin et al. (2003) encouraged participants to take frequent breaks and stopped the assessment temporarily if a participant became too distressed; with a trained clinician available to assess the participant's readiness to continue with participation [34]. In addition to the questionnaire design, a reminder could be included at the beginning of each ACASI session, reminding participants that they are not obliged to answer any question that they find upsetting or inappropriate and may withdrawal from the research at anytime without giving a reason [33]. ACASI administered study questionnaires must be designed with careful consideration of unforeseen harms in recalling past risk behaviors of participants. Designing questionnaires in this manner can help minimize any potential discomfort and remind participants that they do not have to answer questions that cause any discomfort.

There is limited research citing participants' awareness of their contribution to research. Several women acknowledged their participation, including disclosing truthful answers on the ACASI as a contribution to research. This theme is not specific to ACASI but also in other types of data collection. Almeida et al. (2006) found that many of their participants were motivated to participate in human pharmacology clinical research by the potential contribution to the progress of science/medicine. Unfortunately, participation in research does not always translate into contributions to research as a participant may expect [36]. Researchers should clearly describe the intended impact of from the results of their participation. More research is necessary to better understand a participant's perspective on the contribution to research.

Many long lasting and meaningful relationships have the potential to develop between researchers and participants, especially with participants who regularly participation in research. Many HIV-positive individuals are likely to have participated in quantitative and qualitative research experiences as participants in biomedical and social science based research. The rapport that can develop during these research encounters can provide researchers with contextual access to a part of an individual that cannot be described in analysis of data collected with ACASI.

A couple of women mentioned the preference of qualitative FTF interviews versus the computer based self-administered questionnaire using the ACASI. Being able to provide an interviewer with context to a response or receive feedback from an interviewer was important to these participants who felt the absence of personal interaction while using ACASI. Feedback includes probing or asking the participant questions to clarify a response. Probing allows interviewers to pursue the content of responses without stating the dimensions of the response are being taken into account [37]. Probing is an invitation for the respondent to elaborate on his or her response. Qualitative FTF interviews require thinking and conversation by the interviewer (who is or may be viewed as the researcher) and the participant. Qualitative researchers are interested in not only responses, but also context surrounding responses, emotion, and the behavior of an individual [38]. Qualitative interviewers are able to use semi-structured questions and probing to explore the context surrounding the participant's life. FTF interviews can lead to a relationship of mutual benefit; however for the participant there are instances where FTF interviews can make participants experience discomfort [35], just as it has been demonstrated here with this study and with other methods of data collection [32]–[33].

For some women, FTF interviews can be therapeutic [39]; validating a participant's self worth by reinforcing the importance of their story. It also contributes to a researcher's understanding of the participant's complete experience [35]. Due to the stigmatizing nature of some conditions or illnesses (e.g. HIV/AIDS), many participants' rely on this confidential relationship with a researcher; seeing this relationship as a one that will not be violated because of assurance of confidentiality and privacy. Individuals are not able to disclose their condition or circumstantial risk-behaviors because of this stigma see the researcher-participant dyad as an important outlet. ACASI does not allow participant to share their stories or provide context to their experiences or risk behaviors. Participants can find themselves engaging in a socially supportive relationship with the researcher; a relationship that initially commenced with research-only intentions [40].

The participant-researcher relationship could potentially mediate any unintentional harm or discomfort that may result from asking sensitive questions. Qualitative interviews allow for flexibility in the progression of asking and responding to questions that is not available in a self-administered questionnaire or with ACASI. Interviewers can allow respondents to regulate the interview process; encouraging them take their time responding to a question, to recover from disclosing information that may be distressful, or to remind them that they do not have to answer questions that are too personal or cause distress. Probing responses and providing feedback have the potential to validate the worth of respondent's experience and support the respondent as they re-live the experience [39].

Despite this outlet for participants, the possible therapeutic effect of an interview cannot be promised as a benefit, nor can it be the purpose of the interview. Though questionnaires administered using ACASI seemed to allow participants to be more honest, it is not a substitute for the depth of data that can be collected through qualitative research, such as FTF interviews.

Presently, there is limited research examining HIV-positive women's experiences using ACASI in data collection. This analysis explores the experiences of women using ACASI; adding to a limited body of research and suggesting three themes not previously highlighted in published ACASI literature: 1) unintentional harm or discomfort with participation; 2) acknowledgment of contribution to research; and 3) the noted absence of the participant-researcher relationship with ACASI. These three concepts should be further explored in future qualitative research in addition to the further examination of honesty and privacy as it relates to participant experiences with ACASI. ACASI should be considered a data collection modality to minimize the stigma often experienced by HIV-positive women because of its ability to provide privacy, allowing for women to freely disclose risk behaviors and traumatic histories honestly and without inhibition.

One study limitation is the provision of different incentives at each site. Women in Philadelphia received a $10 cash incentive; whereas women in Chicago received a $30 gift certificate. Future research should provide a common amount and type of incentive to eliminate any potential bias introduced by offering different incentives. Our eligibility criteria for participation (e.g. English speaking, women) and the inclusion of two sites introduced selection bias into our study and limit the generalizability of our results. Non-English speaking women may experience the ACASI differently because of language barriers. In this clinical setting, men may have experienced the ACASI differently which could add to the peer-reviewed literature and further substantiate previous findings among HIV-positive men. Future research should examine differences in ACASI experiences across gender but especially among women across racial/ethnic backgrounds, languages, age and socioeconomic status.

Despite our findings on some of limitations of ACASI (e.g. unintentional harm or discomfort and the absence of participant-researcher relationship), we believe that ACASI is an appropriate data collection method for sensitive subjects, particularly for vulnerable women such as HIV-positive women. Combining ACASI with other data collection methods, such as FTF qualitative interviews can strengthen the quality of the data collected and meet the multiple needs of HIV-positive women. These findings not only added to themes to be considered in general methods of data collection but also ACASI-specific themes. ACASI ensures privacy and honesty, may help minimize socially desirable responses, and consequently help participants avoid actual or perceived judgment and improve the quality of HIV behavior risk data. Future research should continue exploration of women's experiences with ACASI through post intervention qualitative interviews.
